# *Protochlamydia* *naegleriophila* as Etiologic Agent of Pneumonia

**DOI:** 10.3201/eid1401.070980

**Published:** 2008-01

**Authors:** Nicola Casson, Rolf Michel, Karl-Dieter Müller, John David Aubert, Gilbert Greub

**Affiliations:** * University Hospital Center and University of Lausanne, Lausanne, Switzerland; † Central Institute of the Federal Armed Forces Medical Services, Koblenz, Germany; ‡Institut für Medizinische Mikrobiologie der Universität Essen, Essen, Germany

**Keywords:** Obligate intracellular bacteria, chlamydia-like organisms, pathogenicity, pneumonia, TaqMan PCR, chlamydiae, parachlamydiaceae, protochlamydia, quantitative PCR, dispatch

## Abstract

Using ameba coculture, we grew a *Naegleria* endosymbiont. Phenotypic, genetic, and phylogenetic analyses supported its affiliation as *Protochlamydia* *naegleriophila* sp. nov. We then developed a specific diagnostic PCR for *Protochlamydia* spp. When applied to bronchoalveolar lavages, results of this PCR were positive for 1 patient with pneumonia. Further studies are needed to assess the role of *Protochlamydia* spp. in pneumonia.

Recently, a *Naegleria* endosymbiont (KNic) was observed but remained uncultivable, precluding precise identification (*1*). We grew a large amount of strain KNic by using *Acanthamoeba* *castellanii,* which enabled phenotypic, genetic and phylogenetic analyses that supported its affiliation as *Protochlamydia* *naegleriophila*. This new ameba-resistant intracellular bacteria might represent a new etiologic agent of pneumonia because it is likely also resistant to human alveolar macrophages *(**2**,**3**)*. Because other *Parachlamydiaceae* were associated with lung infection *(**4**–**6**)*, we assessed the role of *Pr. naegleriophila* in pneumonia by developing a diagnostic PCR and applying it to bronchoalveolar lavages.

## The Study

KNic growth in *A.* *castellanii* was assessed by immunofluorescence with in-house mouse anti-KNic and Alexa488-coupled anti-immunoglobulin antibodies (Invitrogen, Eugene, OR, USA). Confocal microscopy (LSM510; Zeiss, Feldbach, Switzerland) confirmed the intracellular location of KNic and demonstrated its rapid growth within *A. castellanii*. To precisely assess the growth rate, we performed PCR on *A. castellanii*/KNic coculture by using PrF/PrR primers and PrS probe. After 60 hours, we observed an increased number of bacteria per microliter of 4 logarithms (Appendix Figure).

**Figure 1:**
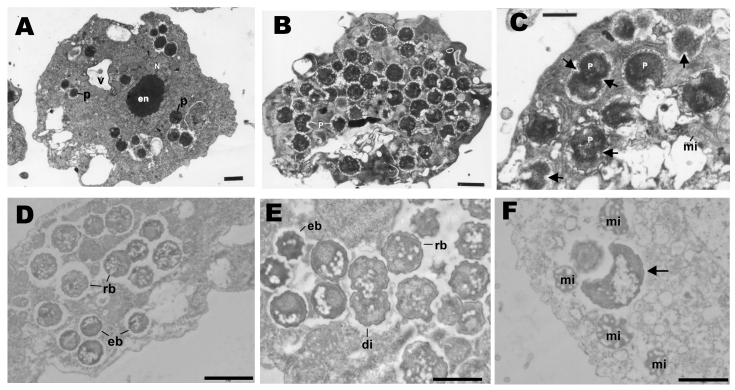
Transmission electron microscopy of *Protochlamydia* *naegleriophila*. A) *Naegleria lovaniensis* trophozoite after transfer of endocytobionts; strain KNic (p) from the original host strain showing 15 coccoid bacteria distributed randomly within the cytoplasm of the host ameba. N, nucleus; en, endosome (karyosome) within the nucleus; v, empty food vacuoles. Magnification ×10,500; bar = 1 μm. B) *N. lovaniensis* trophozoite jammed with numerous endoparasitic stages of *Pr. naegleriophila*. Magnification ×16,800; bar = 1 μm. C) Enlarged detail of *N. lovaniensis* trophozoite with intracytoplasmic stages of *Pr. naegleriophila*. Some stages show binary fission indicated by the fission furrow (arrows). The endoparasites have a wrinkled gram-negative outer membrane rendering a spiny appearance to the endoparasites. Signs of damage are obvious within the cytoplasm of the host ameba. mi, mitochondria. Magnification ×43,500; bar = 0.5 μm. D) *Pr. naegleriophila* within vacuoles of *Acanthamoebae castellanii* ameba 2 days postinfection. Elementary bodies (eb) and reticulate bodies (rb) are visible. Elementary bodies harbor a smooth membrane compared with the reticulate bodies, which have a spiny shape. Magnification ×10,000; bar = 2 μm. E) Enlarged detail of *A. castellanii* trophozoite with intracytoplasmic stages of *Pr. naegleriophila* 3 days postinfection. Binary fission is observed (di). Magnification ×20,000; bar = 1 μm. F) Crescent body (arrow) within *A. castellanii* observed 3 days postinfection. Magnification ×20,000; bar = 1 μm.

*A. castellanii*/KNic and *N. lovaniensis*/KNic cocultures were processed for electron microscopy as described. Ameba filled with bacteria exhibiting 3 developmental stages already described in other *Parachlamydiaceae* *(8)* were observed (Figure 1).

To measure the serologic differentiation index (SDI) between strain KNic and other *Chlamydia*-like organisms, we immunized Balb/c mice to produce anti-KNic antibodies. Purified *Pr.* *amoebophila* (ATCC PRA-7), *Simkania* *negevensis* (ATCC VR-1471), *Parachlamydia* a*canthamoebae* strain Seine, *Waddlia* *chondrophila* (ATCC 1470), *Neochlamydia* *hartmannellae* (ATCC 50802), *Criblamydia* *sequanensis* (CRIB 18), and *Rhabdochlamydia* *crassificans* (CRIB 01) antigens were tested by micro-immunofluorescence against mouse anti-KNic antibodies, whereas KNic antigen was tested with serum against all these different *Chlamydia*-like organisms. SDIs were calculated as described. Serum from mice immunized with KNic showed strong reactivity against autologous antigen (titers of 4,096)*.* Significant cross-reactivity between KNic and *Pr.* *amoebophila* (SDI = 7) and *P. acanthamoebae* (SDI = 10) was observed. Mouse anti-KNic serum did not react with other *Chlamydia*-like organisms (Table 1). Because cross-reactivity between members of the order *Chlamydiales* was proportional to the relatedness between each species, the strong cross-reactivity between KNic and *Pr. amoebophila* supports the affiliation of KNic in the genus *Protochlamydia*.

**Table 1:** Antibody titers and serologic differentiation index (SDI) obtained from reciprocal cross-reactions of mouse antiserum with different *Chlamydia*-like organisms, as determined by immunofluorescence* www.cdc.gov/EID/content/14/1/168-T1.htm

Strains	Antigen titers (SDI)							
	*Pr. naegleriophila*	*Pr. amoebophila*	*Simkania negevensis*	*P. a.* strain Seine	*Waddlia chondrophila*	*Neoclamydia hartmannellae*	*Criblamydia sequanensis*	*R. crassificans*
*Pr. naegleriophila*	4,096 (0)	256 (7)	<4 (14)	512 (10)	4 (20)	16 (14)	<4 (20)	<4 (13)
*Pr. amoebophila*	32 (7)	256 (0)	<32 (5)	1,024 (3)	64 (10)	32 (0)	128 (9)	64 (3)
*S. negevensis*	32 (14)	128 (5)	512 (0)	64 (12)	128 (12)	<32 (12)	512 (8)	256 (3)
*P.* strain Seine	128 (10)	512 (3)	32 (12)	16,384 (0)	64 (19)	64 (12)	<32 (19)	<32 (12)
*W. chondrophila*	32 (20)	128 (10)	32 (12)	<32 (19)	32,768 (0)	<32 (18)	512 (13)	128 (10)
*N. hartmannellae*	32 (14)	128 (0)	<32 (12)	128 (12)	<32 (18)	2,048 (0)	<32 (18)	<32 (11)
*C. sequanensis*	32 (20)	128 (9)	128 (8)	64 (19)	256 (13)	<32 (18)	32,768 (0)	128 (8)
*R. crassificans*	32 (13)	128 (3)	64 (3)	64 (12)	64 (10)	<32 (11)	256 (8)	256 (0)

*Pr., Protochlamydia; *P. a., Parachlamydia acanthamoebae*; *R., Rhabdochlamydia.* Titers highlighted in gray, previously published in (*9*), are provided here because these data were used to calculate SDI between *Pr.* *naegleriophila* strain KNic and the other *Chlamydia*-related bacteria.

Taxonomic position of KNic was further defined by sequencing 16Sr RNA (*rrs*, DQ635609) and ADP/ATP translocase (*nnt*, EU056171) encoding genes. The *rrs* was amplified/sequenced using 16SIGF/RP_2_Chlam primers. The *nnt* was amplified/sequenced using nntF2p (5′-TGT(AT)GAT(CG)CATGGCAA(AG)TTTC-3′) and nntR1p (5′-GATTT(AG)CTCAT(AG)AT(AG)TTTTG-3′) primers. Genetic and phylogenetic analyses were conducted by using MEGA software *(12)*. The 1,467-bp *rrs* sequence showed 97.6% similarity with *Pr. amoebophila*, 91.8%–93.2% with other *Parachlamydiaceae*, and 85.7%–88.6% with other *Chlamydiales*. Based on the Everett genetic criteria *(13)*, KNic corresponds to a new species within the *Protochlamydia* genus because its sequence similarity with *Pr. amoebophila* is >95% (same genus) and <98.5% (different species). Phylogenetic analyses of *rrs* gene sequences showed that KNic clustered with *Pr. amoebophila*, with bootstraps of 98% and 95% in neighbor-joining and minimum-evolution trees, respectively. The 569-bp *nnt* sequence exhibited 91.1% similarity with *Pr. amoebophila*, 65.5%–72.6% with other *Parachlamydiaceae*, and 55.4%–72.6% with other *Chlamydiales*. Phylogenetic analyses of *nnt* sequences showed that KNic clustered with *Pr. amoebophila*. On the basis of these analyses, we propose to name strain KNic “*Protochlamydia* *naegleriophila*.”

We then developed a specific diagnostic PCR for *Protochlamydia* spp. Primers PrF (5′-CGGTAATACGGAGGGTGCAAG-3′) and PrR (5′-TGTTCCGAGGTTGAGCCTC-3′) as well as probe PrS (5′-TCTGACTGACACCCCCGCCTACG-3′) were selected. The 5′-Yakima-Yellow probe (Eurogentec, Seraing, Belgium) contained locked nucleic acids (underlined in sequence above). The reactions were performed with 0.2 μM each primer, 0.1 μM probe, and iTaqSupermix (Bio-Rad, Rheinach, Switzerland). Cycling conditions were as described, and PCR products were detected with ABIPrism7000 (Applied Biosystems, Rotkreuz, Switzerland). Each sample was amplified in duplicate. Inhibition, negative PCR mixture, and extraction controls were systematically tested.

To allow quantification, a plasmid containing the target gene was constructed by cloning PCR products into pCR2.1-TOPO vector (Invitrogen, Basel, Switzerland). Recombinant plasmid DNA quantified using Nanodrop ND-1000 (Witech, Littau, Switzerland) was 10-fold diluted and used as positive controls.

**Figure 2:**
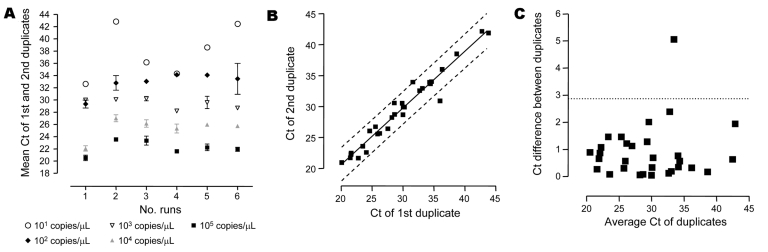
A) Intra and inter-run reproducibility of the real-time PCR assessed on duplicate of plasmidic positive controls performed at 10-fold dilutions from 10^5^ to 10^1^ plasmid/μL during 6 successive runs. Standard deviations show the intra-run reproducibility of the real-time PCR. B) Plots of the cycle threshold (Ct) of first and second duplicates, showing intra-run and inter-run variability of the real-time PCR between duplicates of positive control. 95% confidence interval is shown by the dashed lines. C) Bland-Altman graph showing the difference of Ct of both duplicates according to the mean of the Ct of duplicates. The dashed line shows the 95% confidence interval (i.e., limit of agreement).

The analytical sensitivity was 10 copy/μL (Figure 2, panel **A**). Intra-run variability was good (Figure 2 panel **B**) with a Bland-Altman bias of 0.99 and a limit of agreement of 2.87 (Figure 2, panel **A**). Inter-run variability was low at high concentration, 1.12, 1.71, 0.82, 1.77 cycles for 10^5^, 10^4^, 10^3^, 10^2^ copies/μL, respectively. Inter-run variability was higher at low concentration, 4.22 cycles for 10^1^ copies/μL (Figure 2, panel **A**). Analytical specificity was tested with bacterial and eukaryotic DNA (Table 2). The PCR slightly amplified DNA from *R.* *crassificans*, another *Chlamydia*-like organism. No cross-amplification was observed with any other bacteria or with human cells. The absence of cross-amplification of *P. acanthamoebae* is important because this *Chlamydia*-related bacteria is considered an emerging agent of pneumonia *(**4**–**6**)*.

**Table 2:** Bacterial and eukaryotic DNA used to determine the specificity of the real-time PCR

Bacterial DNA	Source/strain
*Bordetella pertussis*	Clinical specimen
*Chlamydia trachomatis*	Clinical specimen
*Chlamydophila pneumoniae*	ATCC VR-1310
*Criblamydia sequanensis*	CRIB-18
*Enterococcus faecalis*	ATCC 29212
*Escherichia coli*	ATCC 35218
*Gardnerella vaginalis*	Clinical specimen
*Haemophilus influenzae*	ATCC 49247
*Klebsiella pneumoniae*	ATCC 27736
*Lactobacillus* spp.	Clinical specimen
*Legionella pneumophila*	Clinical specimen
*Listeria monocytogenes*	Clinical specimen
*Moraxella catharralis*	Clinical specimen
*Mycobacterium tuberculosis*	Clinical specimen
*Neisseria lactamica*	Clinical specimen
*Neisseria weaveri*	Clinical specimen
*Neochlamydia hartmanella*	ATCC 50802
*Parachlamydia acanthamoebae* strain BN9	ATCC VR-1476
*Parachlamydia acanthamoebae* strain Hall's coccus	ATCC VR-1476
*Protochlamydia amoebophila* strain UWE25	ATCC PRA-7
*Pseudomonas aeruginosa*	ATCC 27853
*Rhabdochlamydia crassificans*	CRIB-01
*Simkania negevensis*	ATCC VR-1471
*Staphylococcus epidermidis*	Clinical specimen
*Streptococcus agalactiae*	ATCC 13813
*Streptococcus mutans*	Clinical specimen
*Streptococcus pneumoniae*	Clinical specimen
*Streptococcus pyogenes*	ATCC 19615
*Waddlia chondrophila*	ATCC VR-1470
Eukaryotic DNA	Source/strain
*Acanthamoebae castellanii*	ATCC 30010
*Candida albicans*	ATCC 10231
Human cells	ATCC CCL-185

We tested 134 bronchoalveolar lavage samples from patients with (n = 65) and without (n = 69) pneumonia and extracted DNA by using a Bio-Rad Tissue Kit. One sample was positive, with 543 and 480 copies/μL. This positive result was confirmed using the 16sigF/16sigR PCR *(13)*, which targets another DNA segment. This sequence exhibited 99.6% (284/285) similarity with *Pr. naegleriophila* strain KNic and 95.1% (269/283) with *Pr.* *amoebophila*. The presence of *Protochlamydia* antigen in the sample was confirmed by immunofluorescence performed using rabbit anti-KNic antibody directly on the bronchoalveolar lavage sample and by ameba coculture (Appendix Figure).

The positive sample was taken from an immunocompromised patient who had cough, dyspnea, and a lung infiltrate. Bronchoscopy examination of the lower respiratory tract showed mucosal inflammation localized at the middle lung lobe. Cytology and Gram stain of the bronchoalveolar lavage showed many leucocytes with macrophages (65%) and neutrophils (23%). Although no antimicrobial treatment was administered prior to bronchoscopy, no other etiologic agent was identified despite extensive microbiologic investigations of bronchial aspirate and bronchoalveolar lavage. Results of Gram stain, auramine stain (for *Mycobacterium* spp.), and silver stain (for *Pneumocystis carinii*) tests were negative. Only physiologic oropharyngeal flora could be grown on sheep-blood and chocolate-bacitracin agars. Cell culture, as well as culture for fungi and mycobacteria, remained sterile. Moreover, results of PCRs specific for the detection of *Legionella pneumophila*, *Chlamydophila* *pneumoniae,* and *Mycoplasma* *pneumoniae* *(15)* were all negative. The patient recovered and remained free of symptoms of acute lung infection during the next 20 months.

## Conclusions

Isolating new species from environmental and clinical samples is important to better define their epidemiology and potential pathogenicity. We defined the taxonomic position of a novel *Naegleria* endosymbiont and proposed its affiliation within the *Protochlamydia* genus as *Pr. naegleriophila* sp. nov. Moreover, we developed a new PCR targeting *Protochlamydia* spp., applied it to clinical samples, and identified a possible role of *Pr. naegleriophila* as an agent of pneumonia.

*Protochlamydia* *naegleriophila* (nae.gle.rio′.phi.la Gr. fem.n. *Naegleria*, name of host cell, Gr. adj. *philos, -a* friendly to, referring to intracellular growth of *Protochlamydia* *naegleriophila* strain KNic within *Naegleria* amebae). The 16Sr RNA sequence (DQ635609) of KNic is 97.6% similar to that of *P.* *amoebophila,* making this organism a member of the genus *Protochlamydia*. KNic does not grow on axenic media *(1)* but grows by 4 logarithms in 60 h within *A. castellanii*. KNic exhibits a *Chlamydia*-like developmental cycle, with reticulate, elementary, and crescent bodies. The reticulate body is about 900 nm and has a spiny appearance similar to that of *P.* *amoebophila* (Figure 2, panel **B**). To be classified within the *Pr. naegleriophila* species, a new strain should show a 16Sr RNA similarity >98.5% *(13)* and similar phenotypic traits.

## Supplementary Material

Appendix Figure A) Growth rate of Protochlamydia naegleriophila within Acanthamoeba castellanii assessed using a specific quantitative real-time PCR. Number of DNA copies present in culture are plotted according to time postinfection. Standard errors of the mean of duplicate experiments are shown. B) Indirect immunofluorescence preformed using rabbit anti-KNic antibody directly on the bronchoalveolar lavage showing the presence of few Pr. naegleriophila strain KNic or C) in clusters of this obligate intracellular bacteria. D) Immunofluorescence performed on amebal coculture showing the presence of Pr. naegleriophila. This strain was lost in subsequent passages.
