# Supplementary figures and images for: Protochlamydia naegleriophila as Etiologic Agent of Pneumonia
Source: Emerg Infect Dis. 2008 Jan;14(1):168–72. doi: 10.3201/eid1401.070980 (PMC2600176; doi:10.3201/eid1401.070980)

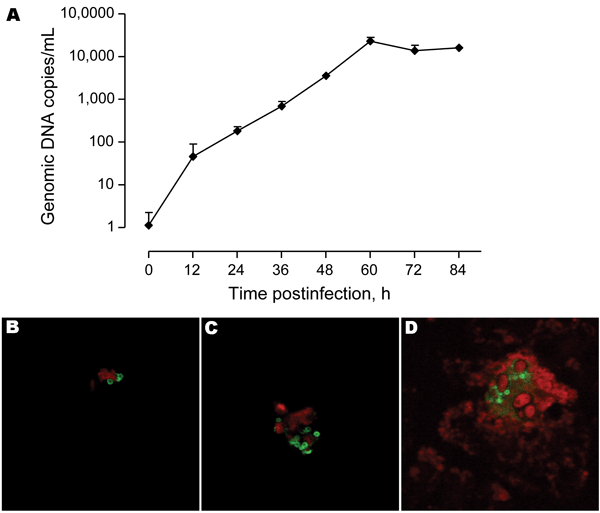

Supplement: Appendix Figure — A) Growth rate of Protochlamydia naegleriophila within Acanthamoeba castellanii assessed using a specific quantitative real-time PCR. Number of DNA copies present in culture are plotted according to time postinfection. Standard errors of the mean of duplicate experiments are shown. B) Indirect immunofluorescence preformed using rabbit anti-KNic antibody directly on the bronchoalveolar lavage showing the presence of few Pr. naegleriophila strain KNic or C) in clusters of this obligate intracellular bacteria. D) Immunofluorescence performed on amebal coculture showing the presence of Pr. naegleriophila. This strain was lost in subsequent passages. [file 07-0980_app-s1.gif]
